# Supplementary material for: Effects of anti-SSA antibodies on the response to methotrexate in rheumatoid arthritis: A retrospective multicenter observational study
Source: PLoS One. 2022 Jul 22;17(7):e0271921. doi: 10.1371/journal.pone.0271921 (PMC9307181; doi:10.1371/journal.pone.0271921)
Supplement: S1 Table — (DOCX) [file pone.0271921.s001.docx]

**S1 table. Patient characteristics at baseline between the patients who achieved LDA based on the DAS28-CRP and those that did not.**

|  | **Missing**  **n (%)** | **Not Achieving LDA group**  **(n = 57)** | **Achieving LDA group (n = 153)** | ***P*** |
| --- | --- | --- | --- | --- |
| Women, n (%) | 0 (0) | 45 (78.9) | 105 (68.6) | 0.17 |
| Age at disease onset, year, median [IQR] | 0 (0) | 61.0 [52.5–69.5] | 60.5 [52.0–71.8] | 0.989 |
| Age at diagnosis, year, median [IQR] | 5 (2.4) | 62.0 [53.0–70.0] | 62.0 [52.0–72.0] | 0.069 |
| Disease duration, months, median [IQR] | 5 (2.4) | 4.0 [1.0–7.0] | 5.0 [2.0–13.0] | 0.033 |
| History of smoking, n (%) | 24 (11.4) | 21 (40.4) | 51 (38.1) | 0.867 |
| IgM-RF positivity, n (%) | 2 (1.0) | 31 (54.4) | 88 (58.3) | 0.64 |
| Anti-CCP antibody positivity, n (%) | 0 (0) | 38 (66.7) | 90 (59.2) | 0.344 |
| Anti-SSA antibody positivity, n (%) | 0 (0) | 14 (24.6) | 18 (11.8) | 0.03 |
| Sicca symptoms, n (%) | 51 (24.3) | 7 (16.3) | 21 (18.1) | 1.000 |
| Diagnosis of Sjögren’s syndrome | 0 (0) | 0 (0) | 2 (1.3) | 1.000 |
| Steinblocker, n (%) | 2 (1.0) |  |  | 0.457 |
| Ⅰ |  | 39 (69.6) | 113 (74.3) |  |
| Ⅱ |  | 14 (25.0) | 23 (15.1) |  |
| Ⅲ |  | 0 (0) | 3 (2.0) |  |
| Ⅳ |  | 3 (5.4) | 11 (7.2) |  |
| Patient VAS score, median [IQR] | 0 (0) | 50.0 [30.0–61.0] | 36.0 [20.0–60.0] | 0.06 |
| Physician VAS score, median [IQR] | 0 (0) | 30.0 [22.0–50.0] | 24.0 [11.0–40.0] | 0.003 |
| Number of tender joints, median [IQR] | 0 (0) | 3.0 [1.0–6.0] | 2.0 [0–4.0] | 0.009 |
| Number of swollen joints, median [IQR] | 0 (0) | 5.0 [2.0–8.0] | 2.0 [1.0–5.0] | 0.001 |
| CRP, mg/dl, median [IQR] | 0 (0) | 0.93 [0.28–2.81] | 0.46 [0.12–1.44] | 0.003 |
| DAS28-CRP, mean ± SD | 0 (0) | 4.01 ± 1.03 | 3.36 ± 1.24 | < 0.001 |
| CDAI, mean ± SD | 0 (0) | 17.38 ± 8.19 | 13.47 ± 9.86 | 0.008 |
| SDAI, mean ± SD | 0 (0) | 19.28 ± 9.07 | 14.66 ± 10.73 | 0.004 |
| Corticosteroid use, n (%) | 0 (0) | 11 (19.3) | 38 (24.8) | 0.466 |
| Corticosteroid dose, mg/day, mean ± SD | 0 (0) | 0.8 ± 2.0 | 0.6 ± 1.3 | 0.466 |
| NSAID use, n (%) | 0 (0) | 28 (49.1) | 57 (37.3) | 0.155 |
| Initial MTX dose, mg/week, mean ± SD | 0 (0) | 7.3 ± 1.1 | 7.2 ± 1.2 | 0.424 |
| csDMARD use, n (%) | 0 (0) | 16 (28.1) | 43 (28.1) | 1.000 |

Data are presented as median [interquartile range], mean ± standard deviation (SD), or n (%).

IgM-RF, IgM rheumatoid factor; anti-CCP, anti-cyclic citrullinated protein; VAS, visual analogue scale; ΔPEG, the numeric difference between patient VAS score and physician VAS score; ΔTSJ, the numeric difference between numbers of tender and swollen joints; CDAI, Clinical Disease Activity Index; SDAI, Simplified Clinical Disease Activity Index; MTX, methotrexate; csDMARD, conventional synthetic disease-modifying antirheumatic drug; CRP, C-reactive protein; DAS28, 28-joint Disease Activity Score; NSAID, nonsteroidal anti-inflammatory drug.
