# Supplementary material for: Effects of anti-SSA antibodies on the response to methotrexate in rheumatoid arthritis: A retrospective multicenter observational study
Source: PLoS One. 2022 Jul 22;17(7):e0271921. doi: 10.1371/journal.pone.0271921 (PMC9307181; doi:10.1371/journal.pone.0271921)
Supplement: Attachment — Submitted filename: Response to Reviewers.docx [file pone.0271921.s002.docx]

**Response to Reviewers**

We appreciate your excellent review of our manuscript. Your valuable comments helped us to improve our manuscript.

***Journal Requirements:***

***When submitting your revision, we need you to address these additional requirements.***

***1. Please ensure that your manuscript meets PLOS ONE's style requirements, including those for file naming. The PLOS ONE style templates can be found at***[***https://journals.plos.org/plosone/s/file?id=wjVg/PLOSOne_formatting_sample_main_body.pdf***](https://journals.plos.org/plosone/s/file?id=wjVg/PLOSOne_formatting_sample_main_body.pdf) ***and h***[***ttps://journals.plos.org/plosone/s/file?id=ba62/PLOSOne_formatting_sample_title_authors_affiliations.pdf***](https://journals.plos.org/plosone/s/file?id=ba62/PLOSOne_formatting_sample_title_authors_affiliations.pdf)

We have modified the full text to match PLOS ONE's style format.

***2. We note that your study used an opt-out consent mechanism. Please state whether the ethics committee approved this consent mechanism.***

According to your requirement, we have added the following sentences; “The ethics committee in each hospital approved this opt-out consent mechanism” in the **Materials and Methods** section (Page 5, Line 91-92).

***3. In your Data Availability statement, you have not specified where the minimal data set underlying the results described in your manuscript can be found. PLOS defines a study's minimal data set as the underlying data used to reach the conclusions drawn in the manuscript and any additional data required to replicate the reported study findings in their entirety. All PLOS journals require that the minimal data set be made fully available. For more information about our data policy, please see*** [***http://journals.plos.org/plosone/s/data-availability***](http://journals.plos.org/plosone/s/data-availability)***.***

***Upon re-submitting your revised manuscript, please upload your study’s minimal underlying data set as either Supporting Information files or to a stable, public repository and include the relevant URLs, DOIs, or accession numbers within your revised cover letter. For a list of acceptable repositories, please see*** [***http://journals.plos.org/plosone/s/data-availability#loc-recommended-repositories***](http://journals.plos.org/plosone/s/data-availability#loc-recommended-repositories)***. Any potentially identifying patient information must be fully anonymized.***

***Important: If there are ethical or legal restrictions to sharing your data publicly, please explain these restrictions in detail. Please see our guidelines for more information on what we consider unacceptable restrictions to publicly sharing data:*** [***http://journals.plos.org/plosone/s/data-availability#loc-unacceptable-data-access-restrictions***](http://journals.plos.org/plosone/s/data-availability#loc-unacceptable-data-access-restrictions)***. Note that it is not acceptable for the authors to be the sole named individuals responsible for ensuring data access.***

***We will update your Data Availability statement to reflect the information you provide in your cover letter.***

After checking again with the Ethics Committee of each hospital, data cannot be shared publicly because the data contain potentially identifying or sensitive patient information. However, data can be available from the Ethics Committee of each hospital (contact via Ethics Committee of Keio University School of Medicine: [med-rinri-jimu@adst.keio.ac.jp](mailto:med-rinri-jimu@adst.keio.ac.jp)) for researchers who meet the criteria for access to confidential data. We have changed the data availability statement.

Changes: The **Data availability statement** section of the manuscript now contains the following sentence:

“Data cannot be shared publicly because the data contain potentially identifying or sensitive patient information. However, data can be available from the Ethics Committee of each hospital (contact via Ethics Committee of Keio University School of Medicine: [med-rinri-jimu@adst.keio.ac.jp](mailto:med-rinri-jimu@adst.keio.ac.jp)) for researchers who meet the criteria for access to confidential data.” (Page 23, Line 313-315)

***Reviewer #1’s comments***

***There were significant differences in patients' VAS, DAS28CRP LDA achievement rate, and history of NSAIDs use, suggesting that SS-A antibody positivity may be a predictor of inadequate treatment response. This conclusion is not fully supported by the data. The study has many limitations, and it is difficult to conclude from the results of this manuscript that it is a risk factor for inadequate treatment response.***

As Reviewer #1 points out, we do not believe that our results can be used to **conclude** that anti-SSA antibodies are a poor prognostic factor for treatment of RA, but our results indicate that anti-SSA antibodies may be a predictor of poor response to conventional MTX therapy, independent of other established poor prognostic factors***.***

***The authors' inadequate treatment response excludes patients who progressed to bDMARD treatment, which is a major limitation in the interpretation of the study results.***

In daily practice, the timing of starting bDMARDs after starting MTX therapy is varied because of the individual response to treatment. In addition, some previous studies have suggested that bDMARDs may be less responsive in anti-SSA antibody-positive patients. We considered including such patients in our study would increase the difference between the two groups. Therefore, we excluded patients who started bDMARDs within 6 months of the start of treatment, considering the variability in timing of treatment and the likelihood of differences. However, the results showed a significantly lower DAS28-LDA rate in the anti-SSA antibody-positive group. We added the sentence about this point in the Discussion section.

Changes: The **Discussion** section of the manuscript now contains the following sentence:

“In daily practice, the timing of starting bDMARDs after starting MTX therapy is varied because of the individual response to treatment. furthermore, some previous studies have suggested that bDMARDs may be less responsive in anti-SSA antibody-positive patients as mentioned above [10,11]. We considered including such patients in our study would increase the difference between the two groups. Hence, we excluded patients who started bDMARDs within 6 months of the starting of the treatment, considering the variability in timing of treatment and the likelihood of differences. However, our results showed a significantly lower LDA rate achieving based on the DAS28-CRP in the anti-SSA antibody-positive group.” (Page 19, Line 241-248)

***The observation period is only 6 months and cannot be referred to as a long-term risk factor.***

We agree with you that our results cannot be referred as a long-term effect for treatment response of RA. We added the sentence about this limitation in the **Discussion** section.

Changes: The **Discussion** section of the manuscript now contains the following sentence:

“Third, Long-term effects of anti-SSA antibodies on the treatment of RA are not clear, since the observation period of this study was 6 months.” (Page 22, Line 291-293)

***Major Revisions Describe the validity of defining inadequate treatment as no significant difference in SDAI and CDAI, but only in patient VAS, NSAIDs use, and DAS28CRP LDA.***

Unlike CDAI and SDAI, DAS28 does not include a Physician VAS. This leads us to believe that variations in Patient VAS may have a relatively large impact on the overall index in DAS28. In addition, a higher weighting factor is assigned to tender joints compared to swollen joints in DAS28 .Therefore, we speculate that the current results show a statistically significant difference only in the LDA achieving rate for DAS28. We added the sentence about this hypothesis in the Discussion section.

As mentioned in the first paragraph of Discussion section, we believe that the difference in NSAIDs use at 6 months was due to the residual pain remaining more in the SSA-positive group.

Changes: The **Discussion** section of the manuscript now contains the following sentence:

“Unlike CDAI and SDAI, DAS28 does not include a Physician VAS [18]. This leads us to believe that variations in Patient VAS may have a relatively large impact on the overall index in DAS28. In addition, a higher weighting factor is assigned to tender joints compared to swollen joints in DAS28. Therefore, we speculate that the current results show a statistically significant difference only in the LDA achieving rate based on the DAS28-CRP.” (Page 20, Line 263-267)

***It should be demonstrated that the lower average dose of MTX in SS-A positive RA does not affect the interpretation of the results.***

As Reviewer #1 pointed out, there was a significant difference in MTX dosage between the two groups in this analysis, but the difference was about 1.0 mg on average. Taking into account the clinical efficacy, we believe it is unlikely that this result led to a treatment response in the SSA-positive group.

In fact, the logistic regression analysis (Table 4) accounting for MTX dosage also retained statistical significance for anti-SSA antibody positivity as an independent poor prognostic factor for achieving LDA in DAS28-CRP. we have added our consideration of this difference in the **Discussion** section.

Changes: The **Discussion** section of the manuscript now contains the following sentence:

“Our results showed that there was a significant difference in MTX dosage between the two groups. However, the difference was about 1.0 mg on average. Considering the clinical efficacy, we believe it is unlikely that this result led to a treatment response in the anti-SSA positive group. In fact, the logistic regression analysis accounting for MTX dosage also retained statistical significance for anti-SSA antibody as an independent poor prognostic factor for achieving LDA based on the DAS28-CRP (Table 4).” (Page 21, Line 268-273)

***For each patient, the change in patient VAS should be plotted before and 6 months after MTX administration.***

According to Reviewer #1’s advice, we have added the new figure as **Fig 2*.*** (Page 12, Line 166-167)

***If the focus is on patient VAS, include patient VAS as an explanatory variable in the logistic regression analysis***

Our hypothesis is that patient VAS should be an intermediate factor regarding anti-SSA antibodies as the prognostic factor and achieving LDA based on the DAS28-CRP as the outcome, therefore, we believe that a multivariate analysis adding this as a variable is not appropriate from a statistical perspective.

***Table 1 should present the incidence of extra-articular complications.***

Patients with extra-articular complications were excluded from this study, and we have added a note about this in the **Materials and methods** section. Thank you for clarifying this point.

Changes: The **Materials and methods** section of the manuscript now contains the following sentence:

“Patients with extra-articular complications were also excluded from this study.” (Page 5, Line 85)

***Reviewere #2’s comments***

***1) In the anti-SSA antibody-positive group, the dose of MTX at 6 months was significantly lower than in the anti-SSA antibody-negative group. The reason why MTX doses were lower in the anti-SSA antibody-positive group should be discussed.***

As we discussed above in our response to Reviewer #1, although there was a significant difference in MTX dosage between the two groups in this analysis, the difference was about 1.0 mg on average. Taking into account the clinical efficacy, we believe it is unlikely that this result led to a treatment response in the SSA-positive group. However, it is true that there was a significant difference, so we have added our consideration of this difference in the **Discussion** section.

Changes: The **Discussion** section of the manuscript now contains the following sentence:

“Our results showed that there was a significant difference in MTX dosage between the two groups. However, the difference was about 1.0 mg on average. Considering the clinical efficacy, we believe it is unlikely that this result led to a treatment response in the SSA-positive group. In fact, the logistic regression analysis accounting for MTX dosage also retained statistical significance for anti SSA antibody as an independent poor prognostic factor for achieving LDA based on the DAS28-CRP (Table 4).” (Page 21, Line 268-273)

***Before multivariable logistic regression analysis, the comparison of variables between patients who achieved LDA according to the DAS28-CRP at 6 months and patients who did not achieve might be informative.***

In response to Reviewer #2, we performed an additional analysis comparing baseline characteristics by dividing patients into two groups: patients who achieved LDA based on the DAS28-CRP at 6 months and those who did not. The results are presented in **S1 Table (Page 27, Line 396-398)**. The results show that patients who did not achieve LDA had higher baseline disease activity, more positivity rate of anti-SSA antibodies, and shorter disease duration.

Changes: The **Supporting information** section of the manuscript now contains the following sentence:

“**Supporting information**

S1 Table. Patient characteristics at baseline between the patients who Achieve LDA based on the DAS28-CRP and those that did not.” **(Page 27, Line 396-398)**.

***3) The disease duration and disease activity at baseline could be the potential poor prognostic factors. Thus, these factors should be also examined by multivariable logistic regression analysis.***

As pointed out by Reviewer #2, the results of S1 Table suggest that disease activity at baseline and disease duration may be potential poor prognostic factors for achieving LDA based on the DAS28-CRP. Considering the sample size in this study, we could not perform a multivariate analysis including all potential prognostic factors. Hence, we performed logistic regression analysis for achieving LDA based on the DAS28-CRP including anti-SSA antibody, IgM-RF, anti-CCP antibody, disease duration, and baseline DAS28-CRP activity after multiple imputations. As a result, the presence of anti-SSA antibodies was still a considerable poor prognostic factor for achieving LDA based on the DAS28-CRP at 6 months (odds ratio: 0.406, 95% confidence interval: 0.174–0.949, P = 0.037) **(Table 5).**

Changes: The **Material and Methods** section of the manuscript now contains the following sentence:

“To explore other potential prognostic factors, we compared baseline characteristics between the two groups that achieved LDA based on DAS28-CRP at 6 months and those that did not. We performed another sensitivity analysis considering those potential prognostic factors.” **(Page 7, Line 126-129)**.

Changes: The **Results** section of the manuscript now contains the following sentence:

“To explore other potential prognostic factors, we compared baseline characteristics between the two groups that achieved LDA based on the DAS28-CRP at 6 months and those that did not (S1 Table). The results show that patients who did not achieve LDA had significantly higher baseline disease activity, more positivity rate of anti-SSA antibodies, and shorter disease duration. Based on these results, we performed logistic regression analysis including anti-SSA antibody positivity, IgM-RF positivity, anti-CCP antibody positivity, disease duration, and baseline DAS28-CRP activity. As a result, the presence of anti-SSA antibodies was still a considerable poor prognostic factor for achieving LDA based on the DAS28-CRP at 6 months (odds ratio: 0.406, 95% confidence interval: 0.174–0.949, P = 0.037) (Table 5).” **(Page 17, Line 208-216)**.

**Table 5. Logistic regression analysis for the rate of achieving low disease activity according to the DAS28-CRP, including baseline DAS28-CRP and disease duration.**

| **Risk factor** | **Odds ratio** | **95% CI** | **P** |
| --- | --- | --- | --- |
| Baseline DAS28-CRP | 0.596 | 0.448–0.792 | < 0.001 |
| Disease duration | 0.998 | 0.993–1.004 | 0.998 |
| IgM-RF positivity | 2.326 | 0.969–5.580 | 0.058 |
| Anti-CCP antibody positivity | 0.384 | 0.150–0.983 | 0.046 |
| Anti-SSA antibody positivity | 0.406 | 0.174–0.949 | 0.037 |

Again, thank you for giving us the opportunity to strengthen our manuscript with your valuable comments and queries. We have worked hard to incorporate your feedback and hope that these revisions enable you to accept our submission.
